# The Effect of Abuse History on Adolescent Patients with Feeding and Eating Disorders Treated through Psychodynamic Therapy: Comorbidities and Outcome

**DOI:** 10.3389/fpsyt.2017.00031

**Published:** 2017-03-02

**Authors:** Annamaria M. Strangio, Lucio Rinaldi, Gianluigi Monniello, Leuconoe Grazia Sisti, Chiara de Waure, Luigi Janiri

**Affiliations:** ^1^Institute of Psychiatry and Psychology, Catholic University, Rome, Italy; ^2^Department of Pediatrics and Child and Adolescent Neurology and Psychiatry, “Sapienza” University of Rome, Rome, Italy; ^3^Institute of Public Health, Catholic University, Rome, Italy

**Keywords:** psychodynamic therapy, adolescents, abuse, addiction disorder, impulse control disorder, feeding and eating disorders

## Abstract

**Objectives:**

The first aim of our study was to compare the characteristics and comorbidities of patients with eating disorders between those who suffered from a childhood abuse and those who did not. Our second aim was to analyze the differences in the outcome of the psychodynamic therapy between abused and not abused patients.

**Methods:**

Twenty-six adolescent patients with eating disorders were assessed. Adolescent were evaluated by a single expert psychiatrist by checklists and questionnaires: EDI 3, SCL 90, BIS11, Dissociative Experiences Scale, Global Assessment of Functioning, SCID II, and CTQ-Self control (SF). According to the results of CTQ-SF (cut-off ≥ 8), patients were divided into two groups: those who had experienced a history of abuse and those who had not. They underwent a psychodynamic psychotherapy and were assessed again after 12 months.

**Results:**

Eleven patients (42.3%) had a history of abuse according to CTQ score. No significant differences were found in abused and not abused patients in their demographic, clinical, and comorbid characteristics (sex, age, type of eating disorder, comorbid impulse control, personality, and addictive disorders). Abused patients showed a significantly higher score in many scale. The psychotherapeutic intervention in patients with a history of abuse resulted only in a significant decrease in symptom checklist-90 (SCL-90) psychoticism dimension (*p* < 0.05), whereas in patients with no history of abuse a significant decrease was found for SCL-90 somatization, obsessive–compulsive and phobic anxiety dimensions, the SCL-90 Global Severity Index, the Eating Disorder Inventory-3 interceptive deficits, and the dissociative experience scale.

**Conclusion:**

Regarding the first aim of our study, we proved that history of abuse is not significantly related to patient comorbidities. Regarding our second aim, history of abuse was related to patient improvement only for psychotic symptoms; whereas patients who had not experienced an abuse improved in a variety of symptoms. Thus, abuse history can be considered as a negative prognostic factor for patients with eating disorders undergoing dynamic psychotherapy. However, this psychotherapy may have a role in preventing early psychotic disorders in patients with and without an history of abuse.

## Introduction

Eating disorders consist of impairment in body image perception and extreme behaviors, such as rejection or desire for food, which debilitate patients in terms of both physical and psychological health.

According to the DSM-5, the complete diagnostic class of eating disorders is named “Feeding and Eating Disorders” and lists: anorexia and bulimia, pica (eating inedible substances), rumination disorder (regurgitation of ingested substances), avoidant/restrictive food intake disorder (lack of interest in the sufficient food intake), and binge eating disorder (1).

In DSM-5, the age of eating disorder onset has been lowered, with a more severe prognosis and the need for a differentiated and complex treatment, which should be specific to developmental disorders of children and adolescents (2). Previous studies have showed a number of similarities between food addiction and other addictive behaviors including activation of specific brain regions and neurotransmitter systems, disrupted neuronal circuitry, and behavioral indicators of addiction, such as continued use despite negative consequences (3). Impulsivity and emotional dysregulation (ED) have a fundamental role in food addiction, as well as both of them play salient roles in drug use disorders (4).

Moreover adolescence is the period of onset of personality disorders (5). According to the literature, obsessive–compulsive and avoidant personality disorders are frequently associated to eating disorder (6).

In adolescence, eating disorders often occurs in comorbidity with other disorders: particularly addictive and impulse control disorders. Recent studies have showed that lots of problems regarding food behaviors, impulse control, and addictive disorders share some common features in adolescent and young people (7).

Other studies have pointed out that sexual and/or physical abuse in childhood exposes the subject to the risk of developing eating disorder (8). According to them, a history of childhood abuse can be associated to eating disorder in 30% of cases (9).

Psychodynamic therapy is frequently used in clinical practice (10). The term refers to an “umbrella” concept for treatments that operate in an interpretive-supportive continuum (11). By interpretive intervention, insight into instincts, affects, object relations, or defense mechanisms may be enhanced. Supportive interventions include fostering a therapeutic alliance, setting goals, or strengthening psychosocial abilities by increasing reality testing or impulse control. The use of more supportive or more interpretive (insight-enhancing) interventions is tailored to the patient’s needs. There is a range of manualized psychodynamic therapies varying in the extent to which they focus on supportive or expressive elements (12). “Psychodynamic psychotherapy has common factors, outlined by Blagys and Hilsenroth, including: focus on affect and expression of emotion, exploration of attempts to avoid distressing thoughts and feelings, identification of recurring themes and patterns, discussion of past experience, focus on interpersonal relations, focus on the therapy relationship, and exploration of wishes and fantasies.” (13).

The efficacy of the psychodynamic psychotherapy in patients with eating disorder has been established especially for anorexia, but promising results have been showed also for all feeding and eating disturbances (14).

Studies on adolescent patients affected by eating disorder have confirmed the efficacy of the psychodynamic therapy; particularly as to improving alimentary symptoms, complying with pharmacological treatments, and implementing the process of adolescent subjectivization (15).

Given the role of childhood abuse in the development of eating disorder and the efficacy of psychodynamic psychotherapy on these disorders even in adolescence, we wanted to investigate how the abuse can influence the psychotherapeutic outcome.

This is relevant also for providing a suitable treatment for abused patients with eating disorder in terms of frequency of sessions, duration and possible integrative approaches.

The first aim of our study was to compare characteristics and comorbidities of two types of patients affected by eating disorder: those who have suffered from a childhood abuse and those who had not. Our second aim was to analyze the psychodynamic therapy outcome in abused and not abused patients after 12 months of treatment. Patients were admitted to the Day Hospital of Psychiatry of the Catholic University and to the Day Hospital for Adolescents of “Sapienza University of Rome.”

We used Eating Disorder Inventory-3 (EDI-3) to investigate the symptoms of eating disorder; Symptom checklist-90 (SCL-90), global assessment of functioning (GAF), and Structured Interview for DSM-IV Axis II (SCID-II) to evaluate psychopathology and comorbid personality disorders; Childhood Trauma Questionnaire-short form (CTQ-sf) to identify possible traumatic events or situations in the history; Barratt Impulsiveness Scale -11 (BIS-11) and Dissociative Experiences Scale (DES) to evaluate the characteristics of complex trauma (16). In fact, many patients with a history of traumatic abuse or neglect suffer from dissociative experiences and impulse dyscontrol which are the most frequent consequences of complex trauma.

## Participants and Methods

### Participants

During a period of 20 months (September 2014–May 2016), patients with eating disorders were observed in both Day Hospitals. The protocol was approved by local IRB and subjects took part in the project after signing an informed consent.

We enrolled 26 adolescents (13–18 years), who received a clinical diagnosis of Feeding and Eating disorders and comorbid Addictive and/or Impulse Control Disorders. They underwent a psychodynamic psychotherapy for 12 months in the Day Hospital setting. Patients with other comorbid psychiatric disorders were excluded from the study. Each patient was scheduled for psychiatric and clinical evaluation at the beginning and at the end of the study.

The diagnosis of feeding and Eating Disorders was formulated at the first interview on the basis of medical history and according to the criteria established by the fifth edition of the Diagnostic and Statistical Manual of Mental Disorders (DSM-5). Likewise, patients were diagnosed as suffering from comorbid Addictive and/or Impulse Control Disorders according to the criteria of DSM-5.

### Study Design and Clinical Sample

We enrolled adolescents who had not yet undergone any psychotherapeutic or pharmacological treatment until the study entry.

After the inclusion and before starting psychotherapy, a single experienced psychiatrist evaluated the patients by the following questionnaires and checklists: eating Disorder Inventory-3, SCL-90, Barratt Impulsiveness Scale -11, DES, GAF, Structured Clinical Interview for DSM-IV Axis II, CTQ-sf.

Patients who reported in one or more of the Childhood Trauma Questionnaire dimensions scores equal to or above the clinical threshold in one or more of the Childhood Trauma Questionnaire dimensions were included in the second one (no childhood abuse).

After the first interview and assessment, patients of both clinical subsamples started a structured psychodynamic psychotherapy, in the hospital environment, for a period of at least 12 months. After 12 months, the same experienced psychiatrist evaluated the patients by the same tests, Childhood Trauma Questionnaire excluded.

### Therapist Selection

Therapists were recruited in both hospital services where the sample was enrolled. Therapists from both services worked according to a common psychodynamic therapy pattern (17) and had been trained in psychotherapy by similar educational programs.

Therapists were in all 10, 7 females and 3 males, with at the least 10 years of clinical activity.

Sessions were once a week, lasting 45 min each, for a total of about 44–48 sessions in 1 year. All therapists supported and followed the work with patients through periodical individual and weekly group supervisions.

Psychodynamic therapy with the adolescents implies the elaboration of original experiences from infancy. According to the Monniello’s model, psychotherapy aims at differentiating the onset of subjectivation from its actual completion. Just as in the neonatal period, intersubjectivity plays a crucial role in adolescence. The therapist function consists in elaborating unconscious and infant contents, and mentally integrating physical sensations of the adolescent newly sexualized body. These objectives are met through dream analysis, free associations, and by encouraging the narrative of the adolescent psychological life.

### Questionnaire and Checklist

Eating Disorder Inventory-3 was released in 2004 and successively implemented to reflect more modern theories related to eating disorders (18). The questionnaire has 91 items divided into 12 subscales based on a 0- to 4-point scoring system. Three scales are specific to eating disorder: drive for thinness (DT), bulimia (B), body dissatisfaction; 9 are general psychological scales not directly relevant to eating disorders: low self-esteem (LSE), personal alienation, interpersonal insecurity (II), interpersonal alienation (IA), interoceptive deficits (ID), emotional dysregulation (ED), perfectionism (P), asceticism (A), maturity fears. The inventory yields six composites: eating disorder risk, ineffectiveness, interpersonal problems, affective problems, overcontrol, general psychological maladjustment. It is also a self-report questionnaire administered in 20 min. We have used the Italian version of EDI-3 (19).

Symptom Checklist-90 is a 90-item self-report symptom inventory designed to measure psychological symptoms and distress during the previous week through 10 primary symptom dimensions and one summary score termed global severity index (GSI) (20). Subjects provide a score from 0 (not at all) to 4 (very much). It is appropriate for individuals from the community, as well as for patients with either medical or psychiatric conditions. The main symptom dimensions include somatization (SOM), obsessive–compulsive, interpersonal sensitivity, depression, anxiety (ANX), hostility, phobic anxiety (PHOB), paranoid ideation (PAR), and psychoticism (PSY), sleep disorders (SLEEP). For each of them the relative score is calculated as the average of questions answered. Scores ≥ 1 are considered significant. We have used Italian version of SCL-90 (21).

Barratt Impulsiveness Scale-11 [BIS-11 by Barratt (22)] is the most widely used questionnaire designed to assess the personality or the behavioral impulsiveness (22). It is composed of 30 items describing common impulsive or non-impulsive (for reverse scored items) behaviors and preferences: attention (A), Cognitive Instability (IC), Motor (IM) (M), Perseverance (P), Self control (SF), Cognitive Complexity (CC), Attentional (A), Motor (IM), and Non-planning (InonPlan). Items are scored on a 4-point scale: rarely/never = 1, occasionally = 2, often = 3, almost always/always = 4. We have used Italian version of Barratt Impulsiveness Scale-11 (BIS-11) (23).

Dissociative Experiences Scale is a psychological self-assessment questionnaire measuring dissociative symptomsand is a screening not diagnostic test for Dissociative Identity Disorder (24) It is made of 28 questions and provides an overall score (cut-off = 30 for Dissociative Identity Disorder) as well as 4 subscale scores. Patients with lower scores may have other post-traumatic conditions. We have used Italian version of DES (25).

Global Assessment of Functioning is used by mental health clinicians and physicians to subjectively rate the social, occupational, and psychological functioning of adults (26). We have used Italian version of GAF (27).

The Semi-structured Interview for DSM-IV Axis II (SCID-II) is designed to diagnose DSM-IV Personality Disorders (28). It consists of open-ended questions to investigate the presence of the 10 DSM-IV Personality Disorders and the two categories included in the Appendix (Passive–Aggressive and Depressive Personality Disorders). We have used Italian version SCID-II (29).

Childhood Trauma Questionnaire-short form is a retrospective, self-reportedscreening measure for traumatic experiences in the infancy with 5 subscales, 3 assessing abuse (emotional, physical, and sexual) and 2 neglect (emotional and physical) (30). Each subscale includes 5 items with a 5-point frequency of occurrence: (1) never true, (2) rarely true, (3) sometimes true, (4) often true, and (5) very often true, scores ranging from 5 (no history of abuse or neglect) to 25 (very extreme history of abuse and neglect). A 3-item Minimization-Denial subscale is designed to identify extreme response bias, specifically respondents’ attempts to minimize their experiences. Other traumatic events that may occur in childhood, such as bereavement or major illness, are not assessed. We have used Italian version of CTQ-sf (31).

### Statistical Analysis

Median and interquartile range (IQR) were used to describe quantitative variables, absolute and relative frequencies of categorical variables. The chi-square test was used to evaluate the association between CTQ (cut-off = 8) and sex, eating, impulse control and addiction disorders. Mann–Whitney test was used to test if age and baseline scores were different in abused and non-abused patients according to CTQ.

In order to test the change from the baseline to 12 months of follow-up, the Wilcoxon signed rank test and the McNemar test were used for quantitative and dichotomous variables, respectively. The effect size was investigated through the Wilcoxon Rank test and phi(φ) for McNemar test (32). Effect sizes were evaluated using the following formulas: $r=Z\text{/}\sqrt{N}\text{ and }\varphi =\sqrt{\left(\chi^{2}\text{/}N\right)}$ and have been interpreted according to Cohen (32). An analysis of covariance (ANCOVA) was performed in order to investigate any significant differences in the effect of intervention between abused and not abused patients; the pre-test scores were used as covariate in order to adjust the analysis. Furthermore, a stratified analysis for patients with or without history of abuse was performed in order to assess the efficacy of the intervention in each group. A *p*-value <0.05 was considered significant, with no correction applied.

## Results

### Subject’s Characteristics at Baseline

Patients’ characteristics are shown in Table 1. The median age was 16; 16 of 26 patients were female. Eleven patients (42.3%) had a history of abuse according to CTQ score, with respect to subscales high median values were found especially for emotional neglect: emotional abuse (median: 15, IQR:10), physical abuse (median: 11, IQR:18), sexual abuse (median: 6, IQR:7), emotional neglect (median: 36, IQR:13), and physical neglect (median:12, IQR:12); 10 out of 26 patients had a binge eating comorbidity.

**Table 1:** **Patients’ demographic and clinical characteristics**.

	Total sample (26)	Abused patients (11)	Not abused patients (15)	*p*-Value
***Sex***				
M	10 (38.5%)	4 (36.4%)	6 (40%)	1
F	16 (61.5%)	7 (63.6%)	9 (60%)	

***Age***				
median	16	16	16	0.458
interquartile range (IQR)	3	3	4	

Anorexia	5 (19.2%)	1 (9.1%)	4 (26.7%)	0.629
Binge eating disorder	10 (38.5%)	4 (364%)	6 (40%)	
Bulimia	2 (7.7%)	1 (9.1%)	1 (6.6%)	
Avoidant/restrictive food intake disorder	9 (34.6%)	5 (45.5%)	4 (26.7%)	

Kleptomania	1 (3.8%)	1 (9.1%)	0	0.346
Conduct disorder	13 (50%)	6 (54.5%)	7 (46.6%)	
Intermittent explosive disorder	3 (11.5%)	2 (18.2%)	1 (6.7%)	
Oppositional defiant disorder	2 (7.7%)	1 (9.1%)	1 (6.7%)	
None	7 (26.9%)	1 (9.1%)	6 (40%)	

Substance abuse/dependence	6 (23.1%)	5 (45.4%)	1 (6.7%)	0.057
Gambling	1 (3.8%)	1 (9.1%)	0	
Internet gambling	7 (26.9%)	2 (18.2%)	5 (33.3%)	
None	12 (46.2%)	3 (27.3%)	9 (60%)	

Avoidant	5 (19.2%)	2 (18.2%)	3 (20%)	1
Dependent	2 (7.7%)	0	2 (13.3%)	0.492
Obsessive–compulsive	4 (15.4%)	1 (9.1%)	3 (20%)	0.614
Passive–aggressive	11 (42.3%)	7 (63.6%)	4 (26.7%)	0.109
Depressive	6 (23.1%)	3 (27.3%)	3 (20%)	1
Paranoid	8 (30.8%)	5 (45.5%)	3 (20%)	0.218
Schizotypal	3 (11.5%)	1 (9.1%)	2 (13.3%)	1
Schizoid	0	0	0	–
Histrionic	0	0	0	–
Narcissistic	2 (7.7%)	2 (18.2%)	0	0.169
Borderline	5 (19.2%)	4 (36.4%)	1 (6.7%)	0.128
Antisocial	2 (7.7%)	2 (18.2%)	0	0.169

Regarding comorbidities: impulse control and addiction disorders were found in 19 (73%) and 14 patients (53.8%), respectively. Thirteen patients presented conduct disorders, 1 (3.8%) had Kleptomania, 3 (11.5%) intermittent explosive disorder, 2 (7.7%) oppositional defiant disorder; 6 (23.1%) substance-related disorders, 1 (3.8%) gambling, 7 (26.9%) internet gambling. One or more personality disorders were found in 76% (20 patients) of our study sample, passive aggressive being the personality disorder most frequently observed (42.3% in the total sample and 63.6% in abused patients). No significant differences were found between abused and non-abused patients with respect to patients’ characteristics (sex, age, eating disorders, impulse control disorders, personality disorders, addictive disorders) even though, as far as addictive disorders are concerned, a trend was observed with abused patients showing more frequently substance abuse/dependence and gambling (Table 1).

### Baseline

**Figure 1:**
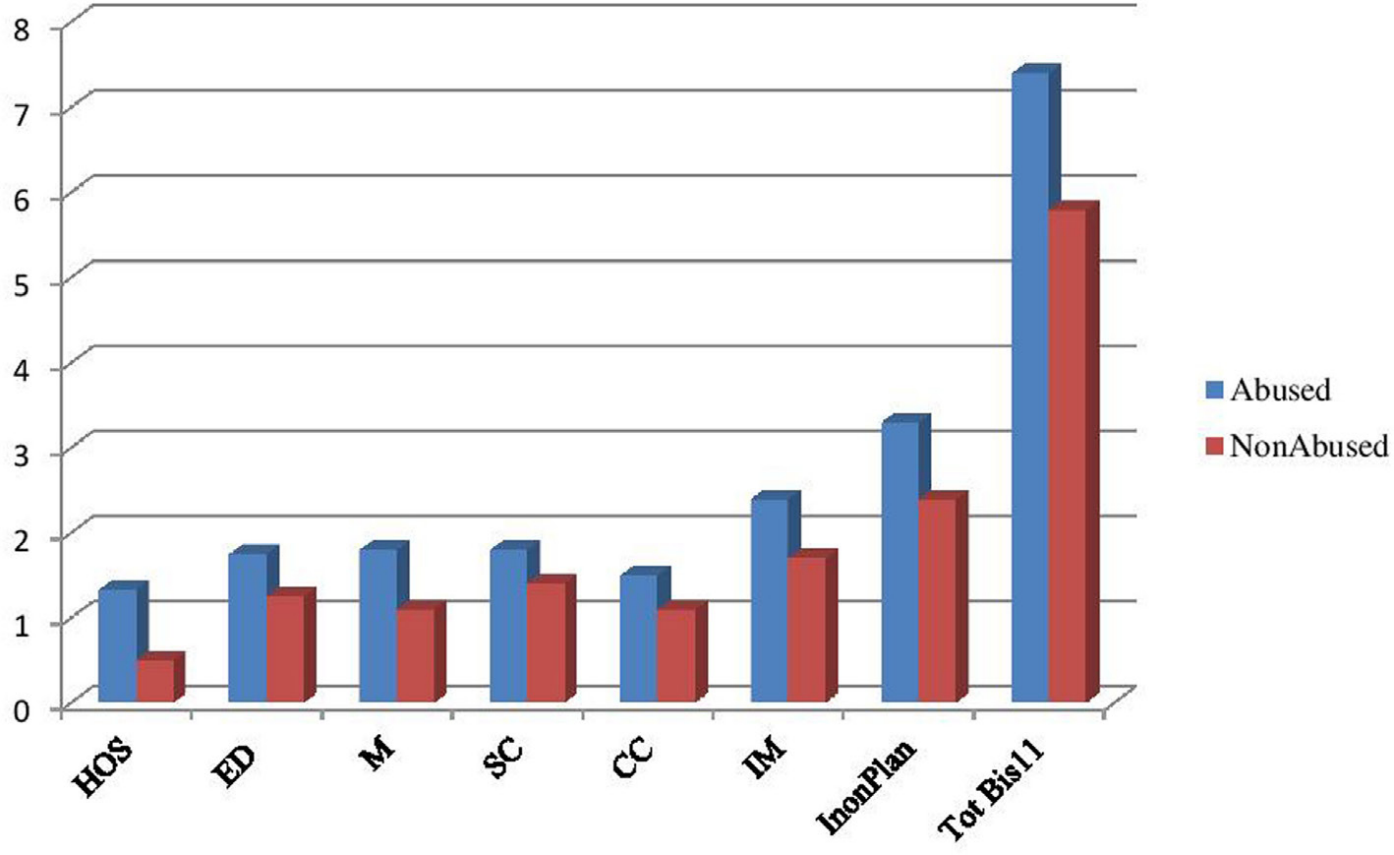
**Variables from baseline to follow-up in the whole sample**.

At the baseline, abused patients showed significantly higher scores in hostility (SCL 90), emotional dysregulation (EDI 3), SF, CC, IM (M), and IM, non-planning, and TotBis11Motor (BIS-11) (Table 2; Figure 1).

**Table 2:** **Baseline test values**.

Symptom variables	Abused patients		Not abused patients		*p*
	Median	IQR	Median	IQR	
**SCL-90**					
SOM	1.50	1.41	1	1.50	0.405
OC	2	1.7	1.3	2	0.550
IS	1.78	2.33	0.56	2	0.287
DEP	1.69	1.92	1.08	1.46	0.311
ANX	1.2	2	0.80	2.2	0.405
HOS	1.33	1	0.50	0.99	**<0.05**
PHOB	0.57	0.85	0.57	1.14	0.465
PAR	1.68	2.66	0.83	1.17	0.349
PSY	0.80	1.8	0.40	1.2	0.076
GSI	1.41	1.75	0.84	1.41	0.233
SLEEP	1.57	1.43	0.71	2	0.132
**EDI-3**					
DT	1.29	2	1.71	3	0.897
B	0.63	2.62	0.87	1.38	0.917
BD	2.60	1.7	1.7	2.8	0.107
PA	2	1.14	1	2.01	0.177
LSE	2.16	2	1.66	2	0.274
II	2.14	1.57	1.71	1.86	0.499
IA	2.14	1.43	1.28	1.86	0.146
ID	1.63	2	1.63	2	0.979
ED	1.75	1	1.25	1	**<0.05**
P	1.33	2	1.16	1	0.639
A	0.86	1.29	1.42	1.57	0.585
MF	1.50	1.88	2	1.25	0.168
**BIS-11**					
A	12	4	11	4	0.228
IC	6	2	5	4	0.289
M	18	6	11	4	**<0.01**
P	7	4	6	3	0.198
SC	18	4	14	5	**<0.01**
CC	15	3	11	4	**<0.05**
IA	19	4	17	7	0.201
IM	24	7	17	3	**<0.001**
Inon Plan	33	5	24	4	**<0.001**
Tot Bis11	74	12	58	10	**<0.001**
**DES**	12.5	33.58	10.71	17.93	0.392
**GAF**	50	10	51	11	0.087

*SCL-90, symptom Checklist-90; SOM, somatization; Obs, obsessive–compulsive; IS, interpersonal sensitivity; DEP, depression; ANX, anxiety; HOS, hostility; PHOB, phobic anxiety; PAR, paranoid ideation; PSY, psychoticism; SLEEP, sleep disorders; GSI, Global Severity Index; EDI-3, Eating Disorders Inventory-3; DT, drive for thinness; B, bulimia; BD, body dissatisfaction; LSE, low self-esteem; PA, personal alienation; II, interpersonal insecurity; IA, interpersonal alienation; ID, interoceptive deficits; ED, emotional dysregulation; P, perfectionism; A, asceticism; MF, maturity fears; DES, Dissociative Experiences Scale; BIS-11, Barratt Impulsiveness Scale-11; A, attention; IC, cognitive instability; IM, Motor (M); P, perseverance; SF, self control; CC, cognitive complexity; A, attentional, IM, InonPlan, non-planning; GAF, global assessment of functioning*.

*Bold font indicates the test abbreviations and significant results*.

Regarding the effects of the psychotherapeutic intervention, results from statistical tests and effect sizes are reported in Table 3. Variables showing a significant change from baseline to follow-up are highlighted in bold. At 12 months, intervention resulted in a significant decrease of score in the variables SOM, OC, ANX, PHOB, PSY, GSI, SLEEP as for SCL-90 scale, in ID, ED, and A as for EDI-3 test, in IC as for BIS-11, and in DES; in almost all these items intervention showed a medium effect size. On the contrary, a statistically significant increase, showing a large effect size, *r* = −0.55, was observed in GAF.

**Table 3:** **Results from statistical tests**.

Test	Baseline		12 months		Test statistics		
	Median	IQR	Median	IQR	*Z*	*p*	*r*
**SCL-90**							
SOM	1	1.31	0.63	0.9	−2.78	**<0.01**	−0.39
OC	1.55	1.80	0.95	1.6	−2.09	**<0.05**	−0.29
IS	1.06	2.06	0.73	1.69	−1.62	0.106	−0.23
DEP	1.16	1.77	0.89	1.83	−1.20	0.230	−0.17
ANX	1.15	2.20	0.55	1.4	−2.35	**<0.05**	−0.33
HOS	1	1.71	0.33	2	−1.46	0.144	−0.20
PHOB	0.57	1	0.14	0.78	−2.81	<**0.01**	−0.39
PAR	1	2.04	0.60	1.99	−2.72	0.204	−0.38
PSY	0.50	1.30	0.25	1.1	−2.76	**<0.01**	−0.38
GSI	0.98	1.46	0.48	1.34	−2.35	**<0.05**	−0.33
SLEEP	1.07	1.75	0.86	1.43	−2.09	**<0.05**	−0.29
**EDI-3**							
DT	1.57	2	1.14	1.74	−1.83	0.067	−0.25
B	0.75	1.41	0.50	1.13	−0.72	0.475	−0.10
BD	2	2.1	1.57	2.2	−1.72	0.085	−0.24
PA	1.28	1.75	1.35	1.31	−0.17	0.869	−0.02
LSE	1.83	2	1.42	1	−1.67	0.094	−0.23
II	1.78	1.64	1.64	1.18	−1.24	0.216	−0.17
IA	1.57	1.61	1.38	0.71	−0.30	0.764	−0.04
ID	1.63	2	1.06	1.45	−1.98	**<0.05**	−0.28
ED	1.44	1	0.99	1.16	−2.13	**<0.05**	−0.30
P	1.25	1	1.33	1	−0.03	0.976	−0.0
A	1.28	1.32	0.98	1.10	−2.04	**<0.05**	−0.28
MF	1.75	1.50	1.37	0.65	−1.34	0.181	−0.19
**BIS-11**							
A	11.50	4	11.50	4	−0.21	0.834	−0.03
IC	6	3	5	2	−2.40	**<0.05**	−0.33
M	12.50	8	13.50	13	−0.32	0.746	−0.04
P	7	3	7	2	−0.16	0.870	−0.02
SC	15	8	13.50	14	−0.38	0.704	−0.05
CC	12	4	13	11	−0.29	0.771	−0.04
IA	18	5	15.50	6	−1.78	0.075	−0.25
IM	18.50	7	20	6	−0.87	0.385	−0.12
Inon Plan	26.50	9	27	10	−0.34	0.731	−0.05
Tot Bis11	65.50	15	63.50	13	−0.73	0.465	−0.10
DES	11.61	23.66	7.22	15.2	−**2.57**	**0.010**	−0.36
**GAF**	51	19	61	10	−**3.95**	**<0.001**	−0.55

*No significant changes were found ion SCID II items*.

With respect to ANCOVA, after adjustment for pre-test values, statistically significant differences in response to treatment between abused and not abused patients were found for the following items of the BIS-11 scale: InonPlan [*F*(1;23) = 7.46 *p* = 0.01], A[*F*(1;23) = 12.08, *p* = 0.002], P[*F*(1;23) = 6.60, *p* = 0.017] with abused patients reporting higher values at follow-up. No further statistically significant differences between the two groups were found.

**Figure 2:**
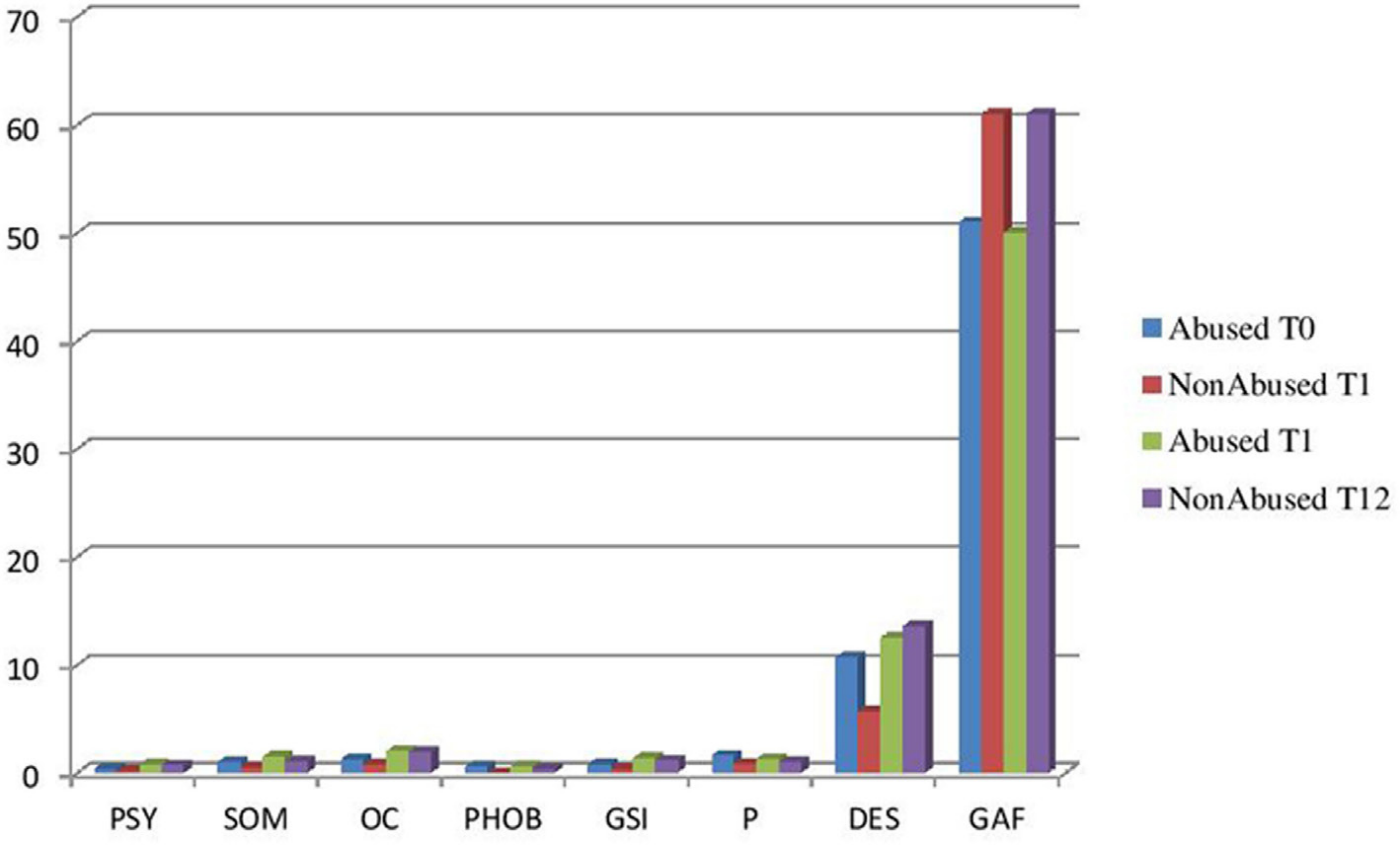
**Stratified analysis variables baseline to follow-up**. Abused/non-abused (*p* < 0.05).

The stratified analysis showed that in patients with history of abuse, intervention resulted only in a significant decrease in PSY, whereas in patients with no history of abuse, a significant decrease was found for SOM, OC, PHOB, GSI, ID, and DES. GAF significantly increased in both abused and not abused patients (Table 4). No significant changes were found in SCID II items in both abused and not abused patients (Figure 2).

**Table 4:** **Stratified analysis**.

Test	Baseline		12 months		Test statistics		
	Median	IQR	Median	IQR	*Z*	*p*	*r*
**(A) Not abused patients**							

**Symptom Checklist-90 (SCL-90)**							
SOM	1	1.50	0.53	1	−2.90	**<0.01**	−0.53
OC	1.3	2	0.80	1.1	−2.05	**<0.05**	−0.37
IS	0.56	2	0.50	1	−1.36	0.173	−0.25
DEP	1.08	1.46	0.46	1.34	−1.50	0.133	−0.27
ANX	0.80	2.2	0.40	0.9	−1.61	0.107	−0.29
HOS	0.50	0.99	0.20	1	−1.57	0.117	−0.29
PHOB	0.57	1.14	0	0.32	−2.20	**<0.05**	−0.40
PAR	0.83	1.17	0.44	1.40	−1.34	0.182	−0.24
PSY	0.40	1.2	0.20	0.4	−1.45	0.142	−0.26
GSI	0.84	1.41	0.40	0.72	−2.10	**<0.05**	−0.38
SLEEP	0.71	2	0.76	0.81	−1.39	0.164	−0.25
**Eating Disorder Inventory-3 (EDI-3)**							
DT	1.71	3	0.86	1.67	−1.48	0.140	−0.27
B	0.87	1.38	0.34	1.11	−0.98	0.328	−0.18
BD	1.7	2.8	0.87	2.1	−1.37	0.172	−0.25
PA	1	2.01	1.54	1.10	−0.51	0.609	−0.09
LSE	1.66	2	1.30	1	−0.51	0.615	−0.09
II	1.71	1.86	1.71	0.90	−0.82	0.410	−0.15
IA	1.28	1.86	1.42	0.57	−1.04	0.300	−0.19
ID	1.63	2	0.88	1.42	−1.99	**<0.05**	−0.36
ED	1.25	1	0.70	0.75	−1.50	0.132	−0.27
P	1.16	1	1.33	1	−0.04	0.972	−0.01
A	1.42	1.57	1	1.09	−1.76	0.078	−0.32
MF	2	1.25	1.4	1.70	−1.33	0.184	−0.24
**Barratt Impulsiveness Scale-11 (BIS-11)**							
A	11	4	11	4	−0.07	0.944	−0.01
IC	5	4	5	2	−1.21	0.227	−0.22
M	11	4	13	5	−1.48	0.138	−0.27
P	6	3	6	1	−0.04	0.972	−0.01
SF	14	5	12	2	−0.28	0.776	−0.05
CC	11	4	12	6	−1.03	0.304	−0.19
IA	17	7	15	7	−0.60	0.550	−0.11
IM	17	3	18	4	−1.80	0.072	−0.33
Inon Plan	24	4	24	6	−0.50	0.614	−0.09
Tot Bis11	58	10	58	8	−0.19	0.850	−0.03
**DES**	10.71	17.93	5.71	6.4	−2.50	**<0.05**	−0.46
**GAF**	51	11	61	9	−3.09	**<0.01**	−0.56

**(B) Abused patients**							

SCL-90							
SOM	1.50	1.41	1.02	1.1	−0.71	0.48	−0.15
OC	2	1.7	1.90	1.7	−0.58	0.56	−0.12
IS	1.78	2.33	1	2.45	−0.85	0.40	−0.18
DEP	1.69	1.92	1.2	1.92	−0.27	0.79	−0.06
ANX	1.2	2	0.90	1.4	−1.75	0.08	−0.37
HOS	1.33	1	1.67	2	−0.47	0.64	−0.10
PHOB	0.57	2.42	0.42	0.71	−1.87	0.06	−0.40
PAR	1.68	2.66	1.54	2.50	−0.42	0.68	−0.09
PSY	0.80	1.8	0.70	1.6	−2.25	**0.02**	−0.48
GSI	1.41	1.75	1.19	1.63	−1.16	0.25	−0.25
SLEEP	1.57	1.43	171	1.29	−1.72	0.09	−0.37
**EDI-3**							
DT	1.29	2	1.14	2.43	−0.97	0.33	−0.21
B	0.63	2.62	1	1	−0.9	0.93	−0.19
BD	2.60	1.7	1.7	2.9	−1.28	0.20	−0.27
PA	2	1.14	1.28	1.66	−1.02	0.31	−0.22
LSE	2.16	2	2	1	−1.82	0.07	−0.39
II	2.14	1.57	1.14	1.42	−0.66	0.51	−0.14
IA	2.14	1.43	1.28	0.71	−1.48	0.14	−0.31
ID	1.51	2	1.12	1.63	−0.61	0.54	−0.13
ED	1.75	1	1.35	1.88	−1.60	0.11	−0.34
P	1.33	2	1	1	−0.15	0.88	−0.03
A	0.86	1.29	0.96	1.72	−1.36	0.17	−0.29
MF	1.50	1.88	1.25	0.32	−0.46	0.65	−0.10
**BIS-11**							
A	12	4	13	3	−0.14	0.88	−0.03
IC	6	2	4	2	−1.90	0.06	−0.40
M	18	6	15	6	−1.21	0.23	−0.26
P	7	4	8	3	−0.43	0.67	−0.09
SF	18	4	19	5	−0.27	0.79	−0.06
CC	15	3	14	8	−0.51	0.61	−0.11
IA	19	4	17	4	−1.89	0.06	−0.40
IM	24	7	23	5	−0.81	0.42	−0.17
Inon Plan	33	5	33	3	−0.05	0.96	−0.01
Tot Bis11	74	12	70	10	−1.36	0.17	−0.29
**DES**	12.5	33.58	13.57	21.8	−1.29	0.20	−0.27
**GAF**	50	10	61	29	−2.53	**0.011**	−0.54

*Bold font indicates the test abbreviations and significant results*.

## Discussion

Our research confirms the correlation between eating disorders and childhood abuse found in the literature (33): 42.3% of our sample reported an abuse history according to CTQ-sf. The most represented eating disorder was the binge eating disorder.

In adolescence, eating disorders are often observed in comorbidity especially with addictive and conduct disorders (34).

Passive–aggressive was the most frequently observed personality disorder. Grover et al. (35), analyzing a group of patients who suffered from a child maltreatment according to CTQ-sf and comparing them with another group without such a history, outlined that many personality disorders, among which the passive-aggressive one, are associated with a child abuse (35).

It is worth pointing out that patients with a history of abuse tend to show more frequent addictive behaviors. Woerner et al. (36) underlined that these patients may have a major tendency to self-punishment, such as using drugs and showing dependent behaviors in general (36).

The history of abuse and/or neglect represents a negative prognostic factor for patients with eating disorder. Harper et al. (37) demonstrated that patients with eating disorder and history of abuse had higher levels of depression, LSE, and worse prognosis (37).

In our sample, at baseline, patients with history of abuse and/or neglect showed more impulsiveness, tendency to acting out, poor mentalization, hostility, and ED; feelings and behaviors related to a state of anger and impulsiveness were observed. This emotional state is likely to derive from early traumatic relationships, either overloaded with emotions or characterized by lack or discontinuous presence of the caregiver. Anger and impulsiveness may lead the patient to actively research sensations.

Jeffrey and Jeffrey (38) assessed the psychological aspects of sexual abuse in female adolescents and evidenced that these patients are at high risk for subsequent acting out behaviors, anxiety, depression, LSE, alcohol and drug abuse or dependence, and sleep and dissociative disorders, eating disorder, emotional numbing, guilt, shame, hyperarousal, and multiple psychiatric disorders (38).

Child sexual abuse is associated with emotion regulation deficits in childhood (39). A not sufficiently protective environment may induce feelings of hostility in the abused child and prevent him to establish positive relationships. The high stress levels suffered by the abused child also hinder his ability to understand and determine cognitive unsteadiness and ED, which are risk factors for eating disorder development (33).

Abused and not abused patients differed as to levels of hostility, ED, and above all, impulsivity, which were higher in the former ones.

Following the treatment, the SCL-90 somatization index and the EDI-3 psychological scale, interoceptive deficit, decreased in the adolescents. One of the main goals of the psychodynamic psychotherapy in individuals expressing a variable level of mental pain through physical symptoms is to attain a condition of subjective appropriation of his own perceptive experiences. In fact, the therapy should provide the adolescent with the ability of mentally representing what he acts and expresses with his body. Through this new awareness the subject can elaborate childhood problems and integrate dissociated parts of himself.

Therapy induced also an improvement of the SCL-90 obsessiveness–compulsiveness index which signals the presence of persistent, irresistible, egodystonic or unwanted thoughts, impulses, and behaviors. Particularly, during the process of subjectivization fantasies, images and anguishes occur together with repetitive and intrusive thoughts. In the therapeutic relationship, the curiosity and the availability of the therapist toward the adolescent vitality are necessary so that patient can afford anguishes and resolve conflicts of childhood. Psychodynamic psychotherapy acts especially by allowing the elaboration of anxious-phobic emotions of patient, improving his introspective abilities, and making him aware of the new somatosensory attitude.

After treatment, all patients improved in other psychopathological domains, including ED, asceticism, IC, dissociative traits, and global functioning.

The two main adaptive tasks of adolescence can be recognized in the psychic integration of pubertal bodily transformations and in the redefinition of identity. These two processes are characterized by strong ambivalence and both generalized and phobic anxiety. Addressing the conflict underlying anxiety is one of the main objectives of the psychodynamic therapy. Other authors, such Cropp et al. (40) examined the results of psychodynamic therapy in adolescents and found an improvement in symptoms and in the psychological general structure (40). Therefore, the improvement in the entire sample of the index of global severity, which reflects the intensity of the subjective discomfort, and the increase of global functioning, which reflects social, working, and psychological functioning, were significant results. The improvement in anxiety indices, ED, asceticism, and IC confirms the efficacy of dynamic therapy in remodeling the adolescent’s mind and allowing him to represent his own emotional states, to take contact with his body and to gain a mentalization attitude, by preventing impulsive manifestations.

However, in the group of patients with a history of abuse and/or neglect, the improvement was much less evident: abused patients remained with higher levels of impulsivity and other psychopathological traits with respect to not abused patients. The improvement of symptoms after psychotherapy was evident in not abused patients in various psychological domains, including impulsivity, dissociation, and global functioning, whereas in abused patients only for psychoticism.

Nevertheless, the index of Psychoticism, including items of seclusion and retirement in addition to relevant symptoms of the schizophrenic dimension, significantly decreased in these patients, which, because of trauma, may be more exposed to deficits in the ego structure and major difficulties in subjectivization. As Rössler et al. (41) underline, child sexual abuse can stand among the risk factors for psychotic disorder. Through dynamic psychotherapy a decrease of possible prodromal schizophrenic symptoms was observed, what is relevant to the prevention of psychotic breakdowns in the presence of a traumatic history. In fact, trauma exerts a very disruptive action on the adolescent’s mental organization (41). In a rater-blinded randomized controlled trial conducted by Weijers et al. (42), patients with a not affective psychotic disorder after 18 months of psychodynamic psychotherapy showed an improvement in positive, negative, anxious, and depressive symptoms (42). The role of psychodynamic psychotherapy could be important in preventing psychotic disorders in adolescents with and without a history of abuse, even though a statistically significant improvement may be clear cut only in the presence of the most severe symptoms.

In conclusion, the limits of our study were the small sample size and the variability determined by a treatment applied by different therapists. However, this latter limit was minimized by the strict adoption of well-established therapeutic rules and by the case supervision by a single supervisor.

Although it is difficult to find patients meeting all the criteria required by the protocol, our target is to enlarge the sample size in further studies.

## Ethics Statement

This study was approved by the Institutional Review Board of Catholic University of the Sacred Heart, Rome, Italy. Subjects over the age of 18 took part in the project after signing an informed consent. For all the participants under the age of 18 who took part in the project, parental consent was also obtained.

## Author Contributions

Full access to all the data in the study and takes responsibility for the integrity of the data and the accuracy of the data analysis: LJ. Study concept and design: AS, LR, GM, and LJ. Acquisition of data: AS. Analysis and interpretation of data: AS, LS, and CW. Drafting of the manuscript: AS. Critical revision of the manuscript for important intellectual content: LR, GM, and LJ. Statistical analysis: CW and LS. Obtaining funding: none. Supervision: LJ.

## Conflict of Interest Statement

The authors declare that the research was conducted in the absence of any commercial or financial relationships that could be construed as a potential conflict of interest.
